# Antimicrobial Resistance and the Alternative Resources with Special Emphasis on Plant-Based Antimicrobials—A Review

**DOI:** 10.3390/plants6020016

**Published:** 2017-04-10

**Authors:** Harish Chandra, Parul Bishnoi, Archana Yadav, Babita Patni, Abhay Prakash Mishra, Anant Ram Nautiyal

**Affiliations:** 1 High Altitude Plant Physiology Research Centre, Hemvati Nandan Bahuguna (H.N.B.) Garhwal University, Srinagar, Garhwal 246174, Uttarakhand, India; hreesh5@gmail.com (H.C.); babita28paatni@gmail.com (B.P.); 2 Department of Microbiology, Bangalore City College, Bangalore 560043, India; bishnoiparul@gmail.com; 3 Department of Microbiology, Institute of Biosciences and Biotechnology, Chhatrapati Shahu Ji Maharaj (C.S.J.M.) University, Kanpur 208024, India; archana25578@gmail.com; 4 Department of Pharmaceutical Chemistry, Hemvati Nandan Bahuguna (H.N.B.) Garhwal University, Srinagar, Garhwal 246174, Uttarakhand, India

**Keywords:** antibiotic resistance, antimicrobial, mechanism of action, plant metabolite

## Abstract

Indiscriminate and irrational use of antibiotics has created an unprecedented challenge for human civilization due to microbe’s development of antimicrobial resistance. It is difficult to treat bacterial infection due to bacteria’s ability to develop resistance against antimicrobial agents. Antimicrobial agents are categorized according to their mechanism of action, i.e., interference with cell wall synthesis, DNA and RNA synthesis, lysis of the bacterial membrane, inhibition of protein synthesis, inhibition of metabolic pathways, etc. Bacteria may become resistant by antibiotic inactivation, target modification, efflux pump and plasmidic efflux. Currently, the clinically available treatment is not effective against the antibiotic resistance developed by some bacterial species. However, plant-based antimicrobials have immense potential to combat bacterial, fungal, protozoal and viral diseases without any known side effects. Such plant metabolites include quinines, alkaloids, lectins, polypeptides, flavones, flavonoids, flavonols, coumarin, terpenoids, essential oils and tannins. The present review focuses on antibiotic resistance, the resistance mechanism in bacteria against antibiotics and the role of plant-active secondary metabolites against microorganisms, which might be useful as an alternative and effective strategy to break the resistance among microbes.

## 1. Introduction

The problem of antibiotic resistance is not limited to the Indian subcontinent only, but is a global problem. To date, no known method is available to reverse antibiotic resistance in bacteria. The discovery and development of the antibiotic penicillin during the 1900s gave a certain hope to medical science, but this antibiotic soon became ineffective against most of the susceptible bacteria. The antibiotic resistance in bacteria is generally a natural phenomenon for adaptation to antimicrobial agents. Once bacteria become resistant to some antibiotic, they pass on this characteristic to their progeny through horizontal or vertical transfer. The indiscriminate and irrational use of antibiotics these days has led to the evolution of new resistant strains of bacteria that are somewhat more lethal compared to the parent strain. Cases of widespread occurrence of resistant bacteria are now very common which leads to many health-related problems [1]. Change in the genetic constitution of these resistant bacteria is so rapid that the effectiveness of common antibiotics may be lost within a period of 5 years [2]. As per the report of the WHO (World Health Organization) resistance was more prevalent in cases of bacterial infections which cause most of the deadly infectious bacterial infections worldwide such as respiratory tract infections, diarrhea, meningitis, syphilis, gonorrhea and tuberculosis [3]. *Staphylococcus aureus* isolated from clinical samples are now showing resistance to more than three drugs and are considered as multiple-drug resistant bacteria [4]. In the case of *Streptococcus pyogenes* isolates, the resistance rates for penicillin are 50% and for erythromycin are 80% globally. The 1539 sputum samples of patients from KwaZulu Natal, South Africa during the period 2005–2006 were screened to study the antibiotic resistance pattern of *M. tuberculosis*; 475 samples were found positive for Tuberculosis (TB) and out of 475 samples, 53 samples were found positive for XDR-TB (Extremely Drug-Resistant Tuberculosis), i.e., they are not susceptible to routine antibiotics used to treat TB [5]. The severity of drug resistance is an alarming situation for scientific and medical professionals, who must search for alternate treatments or develop new drugs to combat drug-resistant bacteria. There is an urgent need of medical assistance for treating these resistant bacteria. The empirical use of antibiotics is one of the factors that contribute to the increase of drug resistance. At times, people use antibiotics without knowing the importance of taking a full course of antibiotics. After taking 2–3 doses of a particular antibiotic, they feel better and then discontinue the course. In comparison to the first line of a drug, the second line or third generation drug are quite costlier and due to the lack of financial assistance these diseases cannot be treated efficiently.

Nowadays, researchers are in search of some novel antimicrobial molecules which have a broad spectrum of activity against both Gram-negative and Gram-positive bacteria without having many or any side effects. They are exploring the variety of medicinal plants which are described in Ayurveda, Charak Samhita, Sushrut Samhita and other literatures available in their respective countries. In the present scenario, some diseases are emerging which are difficult to control by available antimicrobial agents such as XDR strains of *Mycobacterium tuberculosis*, HIV, Hepatitis B, Hepatitis C, Swine flu, Dengue and Japanese encephalitis. There are some antimicrobial drugs which can be used for the treatment of these diseases; however, there are some irreversible side effects associated with them such as liver damage, kidney failure, strokes etc. In recent years, the research on plant-based drugs has increased tremendously and there is some hope seen in certain medicinal plants which can be used for the treatment of these incurable diseases. The aqueous leaf extract of Bhumiamla (*Phyllanthus niruri*) has been reported to have anti-hepatitis B activity. It binds to Hb_S_Ag (surface antigen) and inhibits DNA polymerase required for the multiplication of the Hepatitis virus [6]. *Allium sativum*, *Acalypha indica*, *Adhatoda vasica*, *Aloe vera* and *Allium cepa* are reported to have antituberculosis activity [7].

## 2. Mechanism of Resistance to Antibacterial Agents

**Figure 1:**
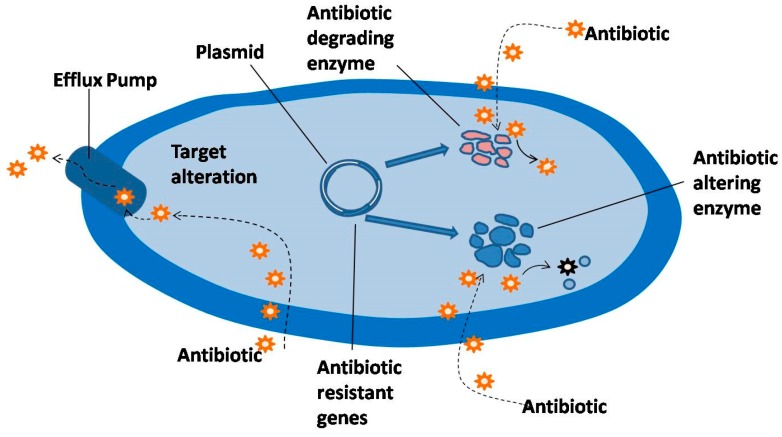
Various ways of resisting the action of antibiotics [8] (Reproduced with permission from IOS Press and D.I. Andersson).

Bacteria become resistant to antimicrobial drugs through different mechanisms (Figure 1; [8]) which are discussed in the following sections.

### 2.1. Antibiotic Inactivation

Some pathogenic microorganisms became resistant to β-lactam antibiotic by modifying the antibiotic or releasing some enzymes such as transferases which inhibit or break down the chemical structure of antibiotics [9]. Plasmids are extrachromosomal material present in the bacteria and carry genes which encode for resistance against certain types of antibiotics. Most of the β-lactam ring-containing antibiotics such as penicillin, ampicillin, amoxicillin, imipenem, piperacillin, ceftazidime etc., become ineffective due to the production of the β-lactamase enzyme which causes hydrolysis of the amide bond in the β-lactam ring [10]. In the case of Gram-negative bacteria, the aminoglycoside group of antibiotics becomes ineffective due to the modification of the antibiotic molecule through phosphorylation, adenylation and acetylation. Over 1000 naturally occurring β-lactamase enzymes have been identified so far [11].

### 2.2. Target Modification

Antimicrobial agents act on a particular site where they bind and alter the normal function; this is called the target site. The bacterial cells become resistant to some antibiotics due to the modification of these target sites. The alteration or modification of the target site may be the result of constitutive and inducible enzymes produced by the bacteria. The pathogenic *Streptococcus* spp. evade the action of MLS_B_ (macrolides, lincosamide and streptogramin B) antibiotics by preventing them from binding to the 50S ribosomal subunit and block protein synthesis by the post-transcriptional modification of the 23S rRNA component of the 50S ribosomal subunit. This is due to the methylation of the N_6_ amino group of an adenine residue in 23S rRNA [12]. A mobile transposable genetic element called SCC*mec*, which contains the *mec*A-resistant gene, is responsible for the resistance in methicillin-resistant *S. aureus* (MRSA) [13].

Vancomycin is a glycopeptide antibiotic that inhibits the cell wall synthesis of bacteria by binding to d-Ala-d-Ala, forming a cap which results in the loss of cross linking in the polypeptide chain. Bacterial species become resistant to vancomycin by changing the usual binding site from d-Ala-d-Ala to d-alanyl-d-serine or d-alanyl-d-lactate at the C-terminus [14]. Aminoglycosides, tetracycline, oxazolidinones, chloramphenicol, fusidic acid, streptogramin, and macrolides are examples of antibiotics that inhibit the growth of bacteria by inhibiting protein synthesis or transcription. The resistance of the bacterial species to these antibiotics is due to the development of mechanisms in which a specific target is modified [15]. The resistance in *Enterococcus* species towards oxazolidinones (linezolid) is due to the effect of mutation in the 23S rRNA, which leads to reduced or feeble affinity for binding [16] and mutation in 16S rRNA causes resistance to aminoglycoside [17]. Resistance developed in *M. tuberculosis* against Streptomycin is a result of mutation in the *rpsL* gene which is responsible for encoding the ribosomal protein S12. Similarly, Fluoroquinolones interfere with the action of DNA gyrase and topoisomerase IV. In the case of *E. coli*, DNA gyrase was the primary target of quinolones while action against topoisomerase IV appeared to be limited, i.e., topoisomerase IV was shown to be a secondary target whereas in the case of *Staphylococcus*, topoisomerase IV was the primary target. In both *E. coli* and *S. aureus*, the resistance against quinolones occurred due to alteration made in either the primary target or secondary target or both.

### 2.3. Efflux Mechanism of Resistance

The intrinsic antibiotic resistance in bacterial genomes is caused by efflux pump proteins encoded by genes that are involved in the maintenance of cellular functions [18]. The information is available in the literature which indicates that active efflux is a mechanism of resistance for almost all antibiotics [19]. Most of the efflux mechanism systems in bacteria are non-drug-specific proteins that identify and expel chemicals, antibacterial agents and structurally unrelated compounds without any changes and degradation of the drug [20]. Expulsion of these antimicrobials or chemicals from the cell results in a low antibiotic concentration which has no or little effect on the growth of bacteria.

The ultrastructure of Gram-negative bacteria revealed that it has a two-layered outer membrane that contains a phospholipid in the inner layer and lipid A moiety of lipopolysaccharide in the outer layer. The penetration and transport of the drug across the outer membrane of Gram-negative bacteria is slightly difficult due to the composition of the outer membrane and transport of the drug is facilitated by porin proteins that form water-filled channels. The entry of antimicrobial molecules through the outer membrane of the bacterial cell may occur either by diffusion through porin or diffusion through the lipid bilayer. The chemical composition of the drug molecule is the most important determinant of the entry mode. For example, chloramphenicol and fluroquinolones penetrated the Gram-negative bacteria with the help of porin [21].

### 2.4. Plasmidic Efflux

The acquisition of new genetic material from other resistant organisms is responsible for the resistance in some bacteria. The transfer of genetic material between the same bacterial species or different species, other than the transfer through the parent to its progeny, is termed horizontal transfer (HGT). Bacterial species may exchange the genetic material through the processes of transformation, conjugation and transduction. These processes of genetic transmission are facilitated by a mobile genetic element, i.e., transposons. Plasmids may carry resistant genes and transmit these to other bacteria (particularly Gram-negative bacteria) through conjugation. During conjugation, pilus form between two bacterial cells, through which the genetic material or plasmid carrying resistant genes are transferred. Enterococcal pheromone-responsive plasmids constitute the mobile genetic element (MGE). The donor cell in the presence of pheromone produces a proteinaceous structure on the cell surface called aggregation substance (AS) which binds to the enterococcal binding substance (EBS) present on the surface of the recipient. A mating channel is formed between the donor and recipient that enables the transfer of the plasmid DNA. After acquiring the plasmid, the recipient stops the production of pheromone and initiates the production of a specific encoded inhibitor peptide which serves to desensitize the bacterial cell to a low level of endogenous and exogenous pheromone produced by the donor. The pheromone-responsive conjugative plasmid system has been most extensively studied for plasmids pAD1, pCF10, and pAM373 from *E. faecalis*. Pheromone-responsive plasmid contributes to the enterococcal phenotype being an important vehicle of antibiotic resistance in *E. faecalis* [22]. In 1998, the plasmid-mediated *qnr* gene which encodes quinolone resistance in bacteria was discovered, the function of which is to protect DNA gyrase from quinolones [23].

Faced with such difficulties and challenges, there is an urgent requirement to search for new antimicrobial molecules or compounds from plant sources which have a broad spectrum of activity against bacterial species as well as having immunomodulatory action. The Indian subcontinent is well known for its traditional knowledge of medicine since time immemorial; a vast number of medicinal plants are described which have immense potential to treat illnesses caused by bacterial species. In India, the use of onion, garlic and ginger as flavoring agents is well documented and well practiced owing to their medicinal value.

## 3. Active Compounds of Plants with Antimicrobial Properties

For the alternative antimicrobial drugs, screening of plants as a source is now being conducted all over the world. Antimicrobial properties in plants are attributed to the presence of active compounds, e.g., quinones, phenols, alkaloids, flavonoids, terpenoids, essential oil, tannins, lignans, glucosinolates and some secondary metabolites (see Table A1 for example). Other antimicrobial agents of plants include the peptides forming their defense systems which are similar to human antimicrobial peptides in structure and function. The comprehensive discussions on various plant-active compounds have been reviewed as follows.

Quinones are compounds with a fully conjugated cyclic Dione structure, such as benzoquinones which consist of two isomers of cyclohexadienedione. These compounds have the molecular formula C_6_H_4_O_2_. The browning reaction in injured or cut fruits and vegetables is due to the presence of Quinones. The dyeing properties of henna (*Lawsonia inermis*) are due to the presence of quinine and it also possesses antimicrobial activity against *Pseudomonas aeruginosa* [24]. Hypericin, an anthraquinone from *Hypericum perforatum*, had general antimicrobial properties and also showed activity against methicillin-resistant and methicillin-sensitive *Staphylococcus* [25].

Alkaloids are phytochemicals commonly found in Angiosperm and rarely found in Gymnosperm. The importance of the medicinal properties of alkaloids first came into existence when morphine was isolated from *Papaver somniferum* which is generally used as pain killer. Caffeine, quinine, cineline, strychnine, brucine, emetine and narcotine are a few examples of alkaloids that have known medicinal value. Berberine is also an example of an alkaloid found in *Berberis* spp., *Cortex phellodendri* and *Rhizoma coptidis* and has antimicrobial activity against *Streptococcus agalactiae*. The mechanism of action of berberine is due to its ability to intercalate with DNA and disrupt the membrane structure by increasing the membrane permeability of bacteria [26]. Hasubanalactam alkaloid isolated from the tubers of *Stephania glabra* has antimicrobial activity against *Staphylococcus aureus*, *S. mutans*, *Microsporum gypseum*, *M. canis* and *Trichophyton rubrum* [27]. The main disadvantage of alkaloids is their toxicity which gives them a marked therapeutic effect in small quantities. That is why the use of alkaloids-based herbal preparation is not frequently used in folk medicine.

Flavonoids are well known phytochemicals that occur in a wide range of plant parts and products mainly in honey, fruits, seeds, vegetables, wines and tea. These phytochemicals are known to have antimicrobial, antiviral, antiallergic and anti-inflammatory properties [28]. The flavonoids such as kaempferol, rutin and quercetin have antifungal properties [29]. A leguminous plant *Lupinus* spp. contains dihydrofuranoisoflavones which showed antifungal activity against *Botrytis cinerea* and *Aspergillus flavus* [30].

Flavones are hydroxylated phenolics containing one carbonyl group (two in quinones), while the addition of a 3-hydroxyl group yields a flavonol. The antimicrobial activity of six flavonoids isolated from *Galium fissurense*, *Viscum album* ssp. *album* and *Cirsium hypoleucum* was shown against extended-spectrum β-lactamase, producing multidrug-resistant bacteria *K. pneumoniae* [31].

Coumarins are phenolic substances made of fused benzene and an alpha pyrone ring [32]. Coumarin isolated from *Angelica lucida* L. is active against the oral pathogens *Streptococcus mutans* and *S. viridians* [33]. Pyranocoumarins isolated from *Ferulago campestris* showed antibacterial activity against nine bacterial strains and the same clinically isolated Gram-positive and Gram-negative bacterial strains [34].

Essential oils are another example of plant secondary metabolites that have compounds with isoprene structure, also known as terpenes, with the typical formula C_10_H_16_. Different types of terpenes are known such as diterpenes, triterpenes and tetraterpenes (C20, C30, and C40), as well as hemiterpenes (C5) and sesquiterpenes (C15). When the compounds contain oxygen as an additional element, they are called terpenoids.

The terpenoids, also known as isoprenoids, are basically a different class of naturally-occurring organic chemicals similar to terpenes. These compounds are multicyclic structures and differ from one another in their basic carbon chains as well as in functional groups. These are the largest group of natural products and can be found in all classes of living things. Plant terpenoids are used for their aromatic qualities. They play a role in traditional herbal remedies and are under investigation for antibacterial, antineoplastic, and other pharmaceutical functions. The characteristic smell of *Eucalyptus*—a smell of cinnamon, cloves, and ginger—is due to the presence of terpenoids. Examples of well-known terpenoids include menthol, citral, camphor, salvinorin A in the plant *Salvia divinorum*, and the cannabinoids found in *Cannabis*.

Terpenenes or terpenoids are active against bacteria, fungi, viruses, and protozoa. *Trichodesma amplexicaule* contains a mixture of terpenoids: β-sitosterol, α-amyrin, lupeol, hexacosanoic acid, ceryl alcohol and hexacosane. The terpenoids extracted from the bark of *Acacia nilotica* have antimicrobial activity against *S. viridans*, *S. aureus*, *E. coli*, *B. subtilis* and *Shigella sonnei* [35]. The essential oil of *Cymbopogon citratus* has moderate activity against *C. albicans* and low activity against *P. aeruginosa*, *E. coli*, *S. aureus* and *T. mentagrophy* [36]. Essential oils are more active against Gram-positive bacteria than Gram-negative bacteria; the possible mechanism of action is membrane permeabilisers.

The term tannin (from *tanna*, German word for oak or fir tree) refers to the use of wood tannins from oak galls and these serve as the source of tannic acid. Tannins have an ability to combine with proteins, resulting in the tanning of animal hides into leather. Chemically, tannin is a large polyphenolic compound containing hydroxyls and carboxyl groups. Tannins present in plant impart astringent (clean the skin and constrict the skin pores) properties and cause a puckering feeling in the mouth when taken orally, e.g., red wine and unripened fruits. The presence of tannins in plants has a defensive role against predation by animals.

It has been well documented that consumption of red wine and green tea, which are good sources of tannins, can cure or prevent a variety of illness by enhancing the immune system [37]. The plant extracts containing tannins cause activation of phagocytic cells and anti-infective actions. Tannins have properties that inhibit the growth and protease activity of ruminal bacteria by binding the cell wall of bacteria [38]. The tannin of Sorghum has antimicrobial activity against *S. aureus*, *Salmonella typhimurium*, *A. niger*, *A. flavus* and *Saccharomyces cerevisae* [39].

Antimicrobial activities of peptides were first reported in 1942. Chemically, antimicrobial peptides have disulfide bonds and are positively charged. These inhibit the growth of bacteria by forming ion channels in the bacterial membrane. The positive charge of antimicrobial peptides binds to negatively charged molecules such as phospholipids, teichoic acid and lipopolysacharide and cause change in the membrane resulting in the death of the bacterial cell. Thionins are the first plant antimicrobial peptides known to kill the plant pathogens; their mechanism of action is to alter the membrane permeability of the microbial cell. Other examples of antimicrobial peptides which have shown promising antimicrobial substance include wheat α-thionin, lipid transfer proteins (LTPs), maltose binding protein (MBP)-1 (isolated from maize) [40] and Ib-AMPs (isolated from *Impateins balsamina*). Fabatin isolated from the fava bean contains 47 peptide residues that have shown antimicrobial activity against *Ps. aeruginosa*, *E. coli*, and *E. hirae* but have not shown any activity against *Saccharomyces* or *Candida* spp. [41]. Pseudothionin (Pth-st1) peptide, isolated from *Solanum tuberosum*, has antifungal and antibacterial activity against *Fusarium solani*, *Clavibacter michiganensis* and *Ps. solanacearum* respectively [42].

Lignans are a group of dimericphenylpropanoids first introduced in 1948 by Howarth. These can be formed by the condensation process of two cinnamyl alcohol/cinnamic acids through the β-carbon of the aliphatic chain. The lignans of some plants are known to have antimicrobial activity. Lignans isolated from *Pseudolarix kaempferi* were reported to have antimicrobial activity against *Candida albican* and *S. aureus* [43]. Dibenzocyclooctadiene lignin isolated from *Schissandra chinensis* inhibits the growth of *Chlamydia trachomatis* and *C. pneumonia* [44].

Glucosinolates are secondary metabolites that consist of sulphur and nitrogen, mainly produced by the Brassicaceae family. Sinigrin is one of the important glucosinolates present in broccoli, mustard and Brussels sprouts and is reported to have antifungal, antimicrobial, anticancer, antioxidant and anti-inflammatory activity [45]. Glucosinolates such as glucoiberverine, glucoiberin and glucoerucin were isolated from the seeds and leaves of *Lobularia libyca* and analysed for antimicrobial activity against *C. albicans* and *Ps. aeruginosa*. It was shown that seed hydrolysates have significant activity against both tested microorganisms [46].

The last few decades have seen a notable shift to a natural health care system and more and more people are resorting to the use of plant-based drugs. Scientific validation of the traditional health care system prevalent in tribal societies, ethnobotanical literature and plants described in Ayurveda, using modern analytical tools, is currently an active area of research. There is growing interest in testing the efficacy of medicinal plants for treating various ailments; also, individual plants as well as combinations of medicinal plants against bacterial species which have become resistant to multiple drugs are being tested. In fact, some of the Allopathic practicing doctors recommend plant-based medicine, especially in the case of liver disease (Jaundice) and other ailments such as joint pain and calculus in the kidney. Recently, Teixobactin was discovered which shows excellent activity against *S. aureus* and *M. tuberculosis* and no resistance is shown to this antibiotic [47].

## 4. Conclusions

Traditional medicines, including plants, have emerged as a boon in medical sciences as they are readily available and have almost no side effects. Plant derivatives have even been proved to cure HIV infection, although only to a certain extent. The identification and isolation of active compounds from the plants is still a challenge for most of the countries rich in plant diversity. *Taxus baccata* is one such example which grows on the upper Himalayas and has active compounds to cure cancer completely by inhibiting the uncontrolled proliferation of the cell. Continuous efforts are being made to explore the plant kingdom in order to find wonder drugs that could save human life from noxious microbial and viral infections such as XDR tuberculosis and HIV infection respectively. Diseases such as Hepatitis B, Hepatitis C, HIV, Swine flu, Dengue, and XDR tuberculosis are a few examples which still pose challenges in allopathic medicines. However, there is evidence which suggests that some medicinal plants are very effective in the treatment of these diseases, as mentioned in this review. The chances of urinary tract infection (UTI) in women are increased during pregnancy. In case a pregnant lady contracts UTI, then the administration of antibiotics is necessary but most of the antibiotics used for the treatment of the infection are contradictory as they may cause severe problems to the fetus. So, it is very difficult for any gynecologist or physician to suggest antibiotics. The most common causal microorganism of UTI is *E. coli* and it can be controlled or eliminated from the urinary tract by use of some medicinal plants such as *Cinnamomum* spp., decoction of Coriander leaf, *Berberis aristata*, *Mimosa pudica*, *Solanum nigrum*, *Raphanus sativus*, *Syzygium aromaticum*, etc. (Table A1). In the last 10–15 years, people have been paying more attention to herbal formulation due to its properties of little or no side effects and action on the root cause of disease. Owing to this, an upsurge in the demand for herbal-based medicines, cosmetics and nutraceuticals has been noticed in India in the last few years.

## Appendix A

**Table A1:** Examples of certain plants and their antimicrobial components.

S. No.	Name of Plants	Antimicrobials	Microbes treated	References
1.	*Acacia nilotica*	Terpenoids, flavanoid, Saponins, Tannins	*S. viridians*, *S. aureus*, *E. coli*, *B. subtilis*, *Shigella sonnei*, *MDR E. coli*, *C. albican*, *K. pneumoniae*	[35,48]
2.	*Allium cepa*	Flavanoid, Polyphenol	MDR *Pseudomonas aeruginosa*, *S*. Typhi, *E. coli*	[49]
3.	*Allium sativum*	Organosulphur compounds (Phenolic compounds), Allicin	*Campylobacter jejuni*, *MDR E. coli*, *C. albican*, *Entamoeba histolytica*, *Giardia lamblia*	[50,51]
4.	*Angelica lucida* L.	Coumarins	*S. viridians*, *S. mutans*	[33]
5.	*Chelidonian majus*	Glycoprotein	*B. cereus*, *Staphylococcus* spp.	[52]
6.	*Cinnamomum* spp.	Cinnamaldehyde (essential oil)	*Legionella pneumophila*, *MDR E. coli*, *C. albican*, *K. pneumoniae*	[53]
7.	*Cirsium hypoleucum*	Flavones	*MDR K. pneumoniae*	[31]
8.	*Curcuma longa*	Curcuminoid (A phenolic compound), turmerone, curlone, Essential oil, curcumins, turmeric oil	*S. typhi*, *E. coli*, *S. aureus*, *B. cereus*, *B. subtilis*, *Ps. aeruginosa*, *B. coagulans*, *A. niger*, *P. digitatum*, Antifungal and antiviral activity	[54]
9.	*Cymbopogan citratus*	Essential oil	*C. albicals*, *Aspergillus flavus*, *A. parasiticus*	[36]
10.	*Galium fissurense*	Flavones	*MDR K. pneumoniae*	[31]
11.	*Hypericum perforatum*	Hypericin (anthraquinone)	Methicillin Resistant *Staphylococcus aureus* and Methicillin sensitive *Staphylococcus*	[25]
12.	*Lawsonia inermis*	Quinones	*MDR Pseudomonas aeruginosa*	[24]
13.	*Medicago sativa*	Saponins, Canavanine	*Enterococcus faecium*, *S. aureus,* Antifungal	[55]
14.	*Mentha longifoilia*	Essential oil	MDR *Staphylococcus aureus*	[56]
15.	*Ocimum basilicum*	Essential oil	MDR *Staphylococcus aureus*, *S.* Typhi, *Aeromonas hydrophila*, *Pseudomonas* spp.	[57]
16.	*Onobrychis sativa*	AMPs (antimicrobial peptides)	*E. faecium*, *S. aureus*	[55]
17.	*Origanum vulgare*	Essential oil	*B. subtilis*, *B. cereus*, MDR *Staphylococcus aureus*	[58]
18.	*Piper longum*	Piperine, Saponin, alkaloid	*MDR B. subtilis*, *Shigella sonnei*	[59]
19.	*Raphanus sativum*	RsAFP2 (Antifungal peptide)	*C. albicans*	[60]
20.	*Rhazy astricta*	Alkaloids and Non alkaloids	*MDR E. coli*, *K. pneumoniae* (*ESBL*), *E. faecium*(*VRE*)	[61]
21.	*Rosmarinus officinalis*	Essential oil	*Streptococcus mutans*	[58]
22.	*Sanguisorba officianalis*	Alkaloids, antimicrobial peptides	*Ps. aeruginosa*, *E. coli*	[52]
23.	*Sorghum* spp.	Tannins	*S.aureus*, *S. typhimurium*, *A. niger*, *A. flavus*, *S. cerevisae*	[39]
24.	*Stephania glabra*	Alkaloids	*S. aureus*, *S. mutans*, *Microsporum gypseum*, *M. canis* and *Trichophyton rubrum*	[27]
25.	*Syzygium aromaticum*	Essential oil, Eugenol	*Streptococcus mutans*, *Staphylococcus aureus*, *Lactobacillus acidophilus*, *Candida albicans* and *Saccharomyces cerevisiae*, MDR *E. coli*, *K. pneumoniae*	[59]
26.	*Vetiveria ziznioides*	Vetivone (vetiver oil)	*Enterobacter* spp.	[62]
27.	*Viscum album*	Flavones	*MDR K. pneumoniae*	[31]
28.	*Zingiber officinale*	Gingerol	*E. coli*, *Enterobacter* spp., *P. aeruginosa*, *Proteus* spp., *Klebsiella* spp., *S. aureus* and *Bacillus* spp.	[49,63]

MDR-Multidrug Resistance, ESBL-Extended Spectrum Beta Lactamase, VRE-Vancomycin Resistant Enterococci.
